# Contributions of NIST/NBS Researchers to the Crystallography of Construction Materials

**DOI:** 10.6028/jres.106.056

**Published:** 2001-12-01

**Authors:** Paul Stutzman

**Affiliations:** National Institute of Standards and Technology, Gaithersburg, MD 20899-8621

**Keywords:** cement, cement clinker, concrete, construction materials, durability, performance, materials science, stone

## Abstract

For more than 100 years, the primary theme underlying the NBS/NIST staff contribution to the crystallography of building materials has been the development of an improved understanding of concrete materials performance. Over that time period, portland cement concrete has become one of the most important of our construction materials for roads, buildings, and other large municipal structures. At the beginning of the 20th century our understanding of portland cement composition, performance, use in concrete, and how the concrete performs in harsh environments was lacking. The efforts of NIST have served to advance construction materials science and technology through the combined efforts of experimental, field study, and theoretical computational materials science. One major achievement in the late 1920s, derived from studies on phase equilibria in cement clinker, allows calculation of potential cement clinker composition. Known as the Bogue calculation, this continues to be an essential tool in cement plant process control to this day. Additionally, contributions of NIST scientists to our knowledge of the chemistry and nature of cement hydration products have been crucial in our understanding of cement hydration and concrete durability. Today, computational materials science is a rapidly developing discipline, and NIST is developing tools incorporating predictive models aided by empirical studies. Examples include a computer-integrated knowledge system for prediction and optimization of performance and life-cycle cost of high performance concrete and the Virtual Cement and Concrete Testing Laboratory. Understanding the relationships between material and performance properties has not been confined only to portland cements. One of the longest running experiments at NIST, the stone test wall, has stood for over 50 years as one of the world’s largest single collections of building stone, and is invaluable for studying weathering effects associated with stone mineralogy and texture. Standards development has also been promoted through participation on ASTM subcommittees on stone, cement, and concrete. The Cement and Concrete Reference Laboratory, established in 1929, continues to provide testing and training for outside laboratories and maintains a historical record of test data on construction materials.

## 1. Introduction

Portland cement concrete has become ubiquitous in its application over the past 100 years as a construction material. Its consumption, at about 5.5 billion tons annually, is second only to water in per-capita demand. Over this time, the flexibility in application and speed of construction afforded by concrete has resulted in it largely supplanting dimension stone as the primary construction material in pavements, homes and high-rise office buildings. It has even been proposed for use in construction of a lunar station! Concrete is an inherently complex material from a chemical and microstructural perspective, but through research it has become a more predictable material, and one with an important future.

**Fig. 1:**
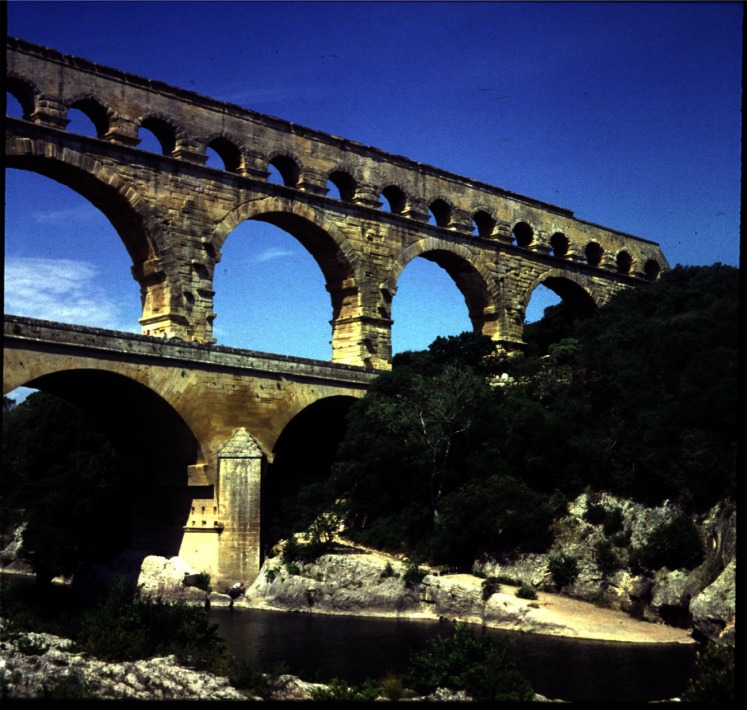
Pont Du Gard, a bridge and aqueduct constructed by the Romans. The aqueduct is lined with cement composed of slaked lime and volcanic scoria [1].

At the beginning of the 20th century, when the National Bureau of Standards (NBS) was formed, the science of portland cement was in its infancy. While calcium silicate-based cements had been in use since Roman times, such as in construction of the Pont du Gard bridge and aqueduct near Nimes in Southern France (Fig. 1), there was a limited understanding of the material properties that affect performance. Little progress in cement technology occurred over the following 1800 years until about 1756, when English engineer John Smeaton discovered that certain clay-rich limestones made superior hydraulic cements compared to those using only pure limestones [1]. The modern understanding of cement chemistry began with the work of Henri Le Chatelier in the late 19th century. His dissertation, “Experimental Researches on the Constitution of Cements and the Theory of Setting,” inspired the 1915 work on high-temperature equilibrium of the system CaO-SiO_2_-Al_2_O_3_ by Rankin and Wright [2] at the Geophysical Laboratory and soon after, studies at NBS in Washington DC.

Improved understanding of the materials science of portland cement has come through increased knowledge of the chemistry and crystallography of the constituent materials. NIST/NBS researchers have played an integral part in developing understanding of portland cement which has, in turn, improved the ability to utilize cement as a construction material. These efforts continue today in the Building Materials Division’s Inorganic Group, which seeks to predict cement and concrete performance properties from a material’s physical properties. Using today’s information technology, the group’s knowledge is embodied in efforts like the Electronic Monograph (http://ciks.cbt.nist.gov/monograph) and the Virtual Cement and Concrete Testing Laboratory (http://vcctl.cbt.nist.gov). This modern-day knowledge is built on the history of the study of building materials, which goes back to the early days of NBS.

In 1917, P. H. Bates and A. A. Klein published the paper “Properties of the Calcium Silicates and Calcium Aluminate Occurring in Normal Portland Cement” [3] which expanded on the research of Rankin and Wright at the Geophysical Laboratory on the constitution of portland cement clinker. This work demonstrated that 3CaO·SiO_2_, or alite, was one of the primary constituents, as postulated by Henri LeChatelier over 30 years previously. This study was also significant in that it was one of the first to address the relationships between cement performance and composition.

Around 1925, the Portland Cement Association (PCA) sponsored a Fellowship at NBS that lasted until the mid-1960s. Researchers at NBS, including those of the PCA Fellowship, were instrumental in developing the foundation of our knowledge of portland cement chemistry through careful study and application of modern scientific techniques. Their fundamental work on the nature and composition of portland cements and portland cement hydration products resulted in the development within The American Society for Testing and Materials (ASTM), of a specification for portland cement (C 150) with five cement classes (types) that are still in use today. In contrast, between 1904 and 1940, ASTM Specification C 9 covered only one all-purpose portland cement [4].

Research efforts of the NBS and PCA fellowship staff helped improve the general knowledge of phase equilibria among the oxides in cement clinker, and also led to additional insight to the limits of solid solution of other oxides in the primary clinker phases. They were instrumental in defining the composition of portland cement clinker with respect to the four primary phases: alite (Ca_3_SiO_5_), belite (Ca_2_SiO_4_), aluminate (Ca_3_Al_2_O_6_), and ferrite (Ca_2_(Al,Fe)_2_O_5_. At the same time, the chemistry and structure of hydration products were the subject of many studies. Their work included the identification and description of key features of the hydration products of portland cements and the reactions between clinker phases and water.

To examine the role of ferric oxide in cements, W. C. Hansen and R. H. Bogue examined fields in the CaO-Fe_2_O_3_-SiO_2_ system, relevant to portland cement, to determine the compounds that exist in equilibrium [5]. Until that time, the role of iron in clinker manufacture, hydration, and setting of portland cements was not well understood.

Lerch and Bogue [6] developed a method for the determination of free lime (CaO) in portland cement. Two prevailing theories were tested on the composition of portland cements: 1) that large volumes of free lime were present and 2) that almost all of the lime occurs in combination with SiO_2_, Al_2_O_3_, and Fe_2_O_3_. Having a rapid method for free lime determination was very helpful in evaluating the extent and combinations of clinkering reactions in the kiln. They also addressed important issues of volume change and soundness that affect durability of concrete structures. The glass content of clinker was investigated by Lerch and Brownmiller by devising a method for its estimation [7], its occurrence in industrial clinkers [8], and its effects upon the heat of hydration of portland cements [9].

**Fig. 2:**
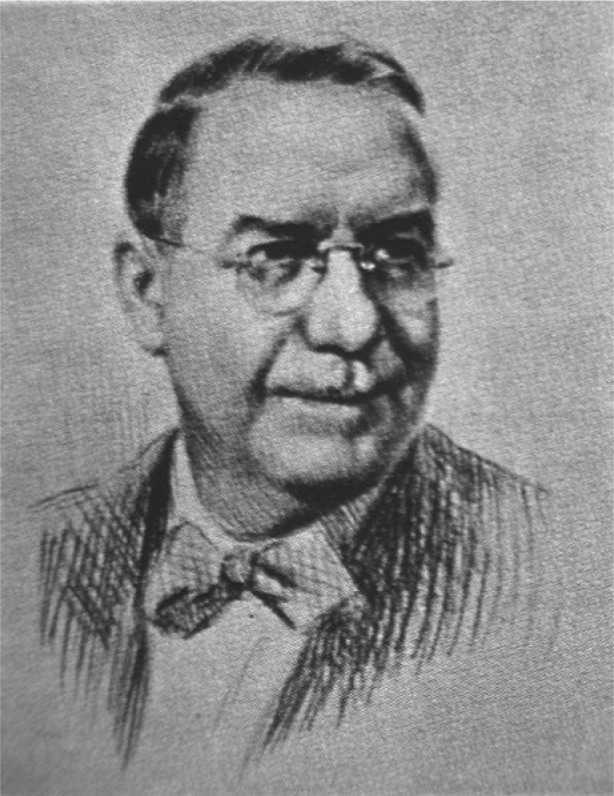
Robert H. Bogue was the first Director of the Portland Cement Association Fellowship at the National Bureau of Standards.

A summary of overall early research in cement technology, including that of the PCA Fellowship, is given by its first Director, R. H. Bogue (Fig. 2), in a 1938 paper at the Symposium on the Chemistry of Cements at Stockholm [10]. Another major achievement was the publishing of the textbook *The Chemistry of Portland Cement* by R. H. Bogue in 1947 [11], and reprinted in 1954. Robert Bogue is perhaps best known for what is called the Bogue calculation [12], whereupon the potential phase composition of a portland cement was estimated from a bulk chemical analysis. This calculation was widely adopted for production of clinker and development of clinker for special applications and is in use today, almost three-quarters of a century later, as part of the ASTM C 150 specification for hydraulic cements. The limitations of this approach were recognized by Bogue, who felt that as the knowledge of portland cement composition evolves so should the means for characterization. Research today in the Building Materials Division is centered on the application of x-ray powder diffraction and microscopy for a more complete and accurate description of portland cement.

Spectroscopy for chemical analysis was employed by Helz [13] and Helz and Scribner [14] for determination of sodium, lithium, and potassium in cements. This method allowed simultaneous analysis of eight elements in cement: Na, K, Li, Mn, Ti, Mg, Fe, and Al. Additional refinements in chemical analysis came with the development of flame photometry and its application in the late 1940s for analysis of sodium and potassium by Eubank and Bogue [15].

Early applications of x-ray crystallography using both single crystal and powder diffraction, as well as electron microscopy, were utilized to better understand the crystal chemistry of cement clinker compounds. The clinker constituents are of commercial interest as they are hydraulic, capable of reacting with water and hardening. The hydration products of portland cement and water, a poorly-crystallized calcium-silicate-hydrate, calcium hydroxide, and sulfoaluminates, are responsible for the strength in portland cement concrete.

X-ray diffraction (XRD) played an important role in the early years of the PCA Fellowship. All the primary clinker phases were synthesized from pure materials, and XRD was used to measure the powder diffraction patterns, which were then compared to those in the patterns of commercial cement clinkers [16]. In 1927, E. A. Harrington was the first researcher to apply x-ray diffraction analysis in the study of clinker constituents [17]. Confirmation of alite as a clinker constituent using x-ray powder diffraction was made by researchers C. W. Hansen and L. T. Brownmiller [18]. X-ray powder diffraction was also used to examine the nature of what was called the “glassy phase” identified by an optical examination [19]. In what was probably an early application of quantitative x-ray powder diffraction analysis, E. A. Harrington developed a method for the study of cements [20].

In 1960, The National Bureau of Standards hosted the 4th International Symposium on the Chemistry of Cement, which drew 271 attendees from 34 countries around the world. The second Director of the PCA Fellowship, Fred Ordway, summarized the knowledge of clinker phase crystal structures at this meeting. He also worked on developing a high-temperature powder camera based upon his earlier work constructing a high-temperature stage for optical microscopy. His other contributions included a structure refinement of tricalcium aluminate, a principal clinker phase, using x-ray diffraction. At the Washington Symposium, D. K. Smith reported on thermal transformations of dicalcium silicate [21]. With at least four polymorphs known to occur, the *β*→*γ* transformation was considered responsible for clinker disintegration phenomena known as dusting. His work also examined calcium aluminates and the chemistry and structure of the ferrite solid solutions of industrial clinkers.

In the late 1970s Geoffrey Frohnsdorff, the Building Materials Division Chief, promoted the formation of a task group on quantitative x-ray powder diffraction under ASTM C 1.23, Subcommittee on Compositional Analysis. This task group was chaired by Leslie Struble and later Paul Stutzman of the Building Materials Division and sought to develop a standard test method for phase abundance analysis of cements using quantitative x-ray powder diffraction. Today, ASTM standard C 1365 [22] provides a method and guidelines for those using x-ray powder diffraction for quantitative phase abundance analysis.

**Fig. 3:**
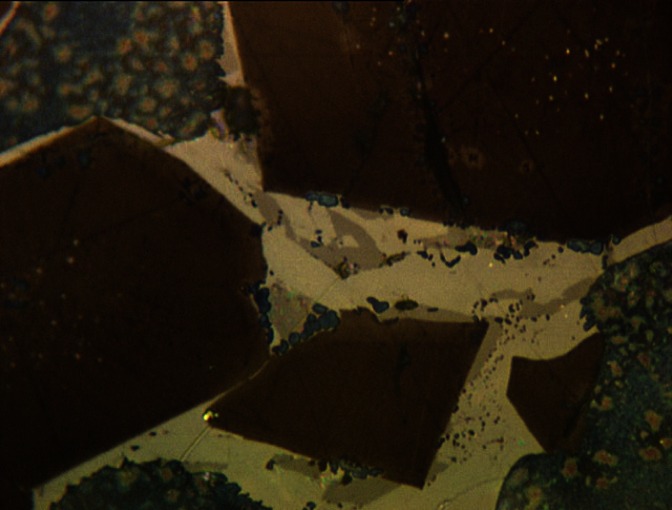
Reflected light microscope image of NIST Reference Clinker 2688 showing alite (brown), belite (blue), ferrite (white), and aluminate (gray) with a field width of 100 μm. Note: Colors shown only in online version.

The optical microscope is a primary tool for the cement chemist for the study of clinker (Fig. 3). Analyses are performed using polished, etched sections using a reflected light microscope, or with thin sections using a petrographic microscope. Early NBS researchers contributed greatly in developing the role of microscopy in cement research and cement manufacturing facilities.

Harold Insley helped to develop the application of reflected light to a microscopic study of polished, etched clinker [23]. Insley also devised a scheme for classification of *β*-2CaO SiO_2_ (belites) polymorphs and through observation of distinct striation patterns identified the occurrence of ferrite (4CaO·Al_2_O_3_·Fe_2_O_3_), an interstitial, solid solution phase in clinker. The confirmation of the occurrence of tetracalcium aluminoferrite as the iron-bearing phase in clinker came with the x-ray identification by W. C. Hansen, L. T. Brownmiller, and R. H. Bogue [24,25,26]. Work by Insley [27] and Howard McMurdie [28,29] investigated the nature and occurrence of the ferrite solid solution. T. F. Newkirk and R. D. Thwaite, examined the range of composition of the ferrite phase and the relationships between bulk composition, temperature, and solid solution composition [30].

The other interstitial phase significant in portland cement performance, tricalcium aluminate, 3CaO·Al_2_O_3_, was also studied by Insley and McMurdie [31] and, CaO·K_2_O·Al_2_O_3_ by L. S. Brown [32] and 8CaO·Na_2_O·3Al_2_O_3_ by L. T. Brownmiller and R. H. Bogue [16,33,34]. Their studies emphasized the optical and chemical properties of the aluminates as well another interstitial phase that was little understood and termed the “glassy phase”.

McMurdie and Insley [35] studied the system CaO-MgO-2CaO·SiO_2_-5CaO·3Al_2_O_3_, important in the developing understanding of the calcium silicates that form the bulk of an industrial clinker. F. P. Hall and H. Insley [36] compiled phase diagrams for silicate scientists that served to aid knowledge of high-temperature phase equilibria and understanding of the nature of clinker compositions.

The desire for improved accuracy in bulk phase composition determinations generated renewed interest in optical microscopy in the late 1970s. ASTM C 1.23 created a Task Group on microscopy, chaired by Paul Stutzman of the Building Materials Division, that developed a standard test method for optical microscopy of portland cement clinker in 1996 [37]. Both the optical and x-ray test method development was facilitated by the availability of a set of reference cement clinkers from the Standard Reference Materials Program at NIST prepared in the late 1980s.

## 2. Hydration and Hydration Products

*Hydration* is a term used by cement chemists to describe conversion of anhydrous cement and water to hardened hydration products. A number of terms are commonly used in reference to this process. *Setting*, where the mixture becomes stiff but does not have appreciable strength, occurs after a few hours. *Hardening* progresses more slowly but is the period with significant gain in mechanical strength. *Curing* is a process where hydration is allowed to proceed [38]. The nature of the hydration products, the conditions of formation, and the environment of exposure are very important as they affect development of strength and durability of a structure.

Bates and Klein in 1917 [3] investigated the hydraulic properties and strength development of the primary cement compounds, the nature of their hydration products, and the influence of bassanite (plaster of Paris) in improving early-age strength. Klein and Philips [39] hydrated calcium aluminates with steam at atmospheric and at high pressure in an autoclave to examine the compositions and textures of the hydration products and the role of gypsum as a retarder. Later, Bates [40] found that calcium sulfate was not an effective retarder of set in the absence of Ca(OH)_2_ and alite. These studies demonstrated that alite sets and hardens like portland cement and aluminate reacts violently with a great deal of heat but has no hydraulic properties, alone or with gypsum. However, when aluminate was added in small quantities to alite mixtures, the resulting products exhibited an increase in strength. Control of the aluminate reaction through addition of gypsum was studied by Lerch [41].

Flint, Wells, McMurdie, and Clarke studied the system CaO-Al_2_O_3_-H_2_O in relation to hydration products of portland cement [42,43,44], and their influence on concrete durability in aggressive sulfate-rich environments [45]. This work continued with a host of studies that sought to better understand hydration reactions, hydration products, the exothermic nature of the hydration reactions, environmental influence on the degradation of hydration products, and relationships between strength and structure of the hydration products [46,47,48,49,50,51,52,53,54,55].

Application of new and old analytical instrumentation continued with Ward utilizing the petrographic microscope to study hydration products [56]. Hunt characterized calcium silicates and their hydration products by IR spectroscopy [57,58]. R. L. Blaine experimented with an early application of NMR to study cements and their hydration products [59]. This was precipitated by his earlier findings that water appeared to be in both the adsorbed and bound state [60]. X-ray powder diffraction studies continued with McMurdie and Flint collecting data on hydration products of calcium silicates [61]. Other studies examined the lime, alumina, water system at various temperatures [62,63,64,65,66,67,68].

## 3. The Cement and Concrete Reference Laboratory

In the early part of the 20th century, various organizations, including The National Bureau of Standards, the U.S. Army Corps of Engineers, the American Society of Civil Engineers, ASTM Committee C 1 on Cement, and the Portland Cement Association, began to standardize the specifications and methods for testing portland cement. This eventually lead to the establishment of the Cement Reference Laboratory (CRL) in 1929 at NBS. Inspection of laboratories was designated as the primary CRL activity. Later, inspections were expanded to include concrete testing. ASTM Committee C 9 on Concrete and Concrete Aggregates became a joint sponsor in 1958 and the name Cement and Concrete Reference Laboratory (CCRL) was adopted in 1960. The CCRL portland cement proficiency sample program for interlaboratory testing distributed their first sample in 1936. They continue to distribute a pair of chemical and physical test samples of portland cement twice a year and have maintained an invaluable historical record of these data.

## 4. Recent Developments

Computational materials science has developed as a scientific discipline powered by the revolutionary advances made in computing power. The major application of this discipline has been to random materials, where analytical approaches are insufficient [70]. Concrete is a random material over length scales ranging from nanometers to meters [69]. Along with this growth in modeling has come an increased emphasis on the careful experimental measurement of concrete microstructure and properties, based on fundamental materials science. An electronic monograph was created by Group Leader Edward Garboczi to disseminate the Building Materials Division work in the computational materials science of concrete. The Group represents a primary center for the computer modeling of the microstructure and properties of concrete as well as experimental measurements of microstructure and properties [70].

Currently, a program titled Partnership for High-Performance Concrete Technology is a major product of the Building Materials Division [71]. Under the direction of Building Materials Division Chief Geoffrey Frohnsdorff, and in partnership with industry, this project’s goals are to promote the development of high-performance concrete in all construction through development of a computer-integrated knowledge system. Called HYPERCON, this knowledge system will incorporate verified multi-attribute models for prediction and optimization of the performance and life-cycle cost of high-performance concrete. Two projects within this program involve characterization of cements and computer simulation of their hydration and microstructure development.

Knowledge of the true phase composition and texture is an initial step in understanding cement performance. One major task is developing and testing of improved methods for characterization of portland cements. As demonstrated by Harrington in the 1920s [17], one means for direct analysis is through powder diffraction analysis. Today, application of Rietveld refinement of cement powder diffraction data has provided both a means to extract detailed structural and chemical information on the constituent phases as well as a means to quantify phase abundance [72]. Backscattered scanning electron microscopy has allowed researchers to image and characterize a ground portland cement composition and texture; a task not possible using only light microscopy. One of the goals of the HYPERCON project on characterization is to build a database of compositional and textural data of North American cements using cements from the CCRL test program [73,74,75]. Having microstructure and composition derived from direct-determination of chemical, textural, and phase composition data will allow exploration of material property-performance property relationships. This knowledge base should facilitate the acceptance of new methods for compositional analysis within ASTM C 01 on cement.

**Fig. 4:**
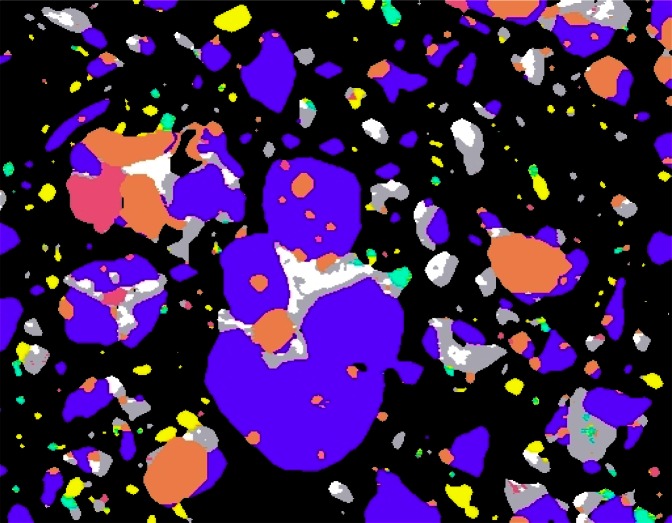
Pseudo-colored scanning electron microscope image of portland cement shows the multiphase character and particle shapes resulting from the grinding of clinker and gypsum with alite (puple), belite (tan), ferrite (white), aluminate (silver), periclase (pink), alkali sulfate (green), and gypsum (yellow). CCRL Cement 135, field width: 150 μm. Note: Colors shown only in online version.

**Fig. 5:**
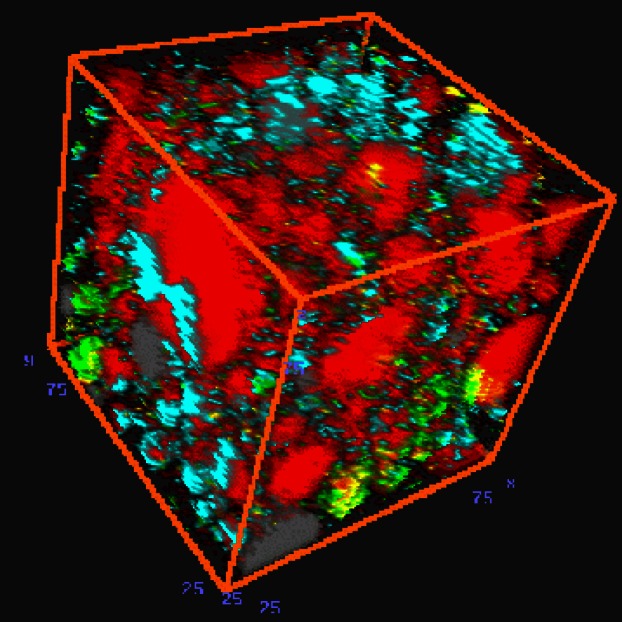
Three-dimensional rendition of cement is based upon experimental (SEM imaging) and computational techniques have been developed to characterize the multi-phase spatial structure [76]. Note: Colors shown only in online version.

Testing and development of cements and concrete is time consuming and costly. To address these difficulties, Dale Bentz has developed a web-based virtual laboratory (Figs. 4 and 5). This is intended for evaluating and optimizing cement-based materials based upon a computer model for the hydration and microstructure development of cement-based systems, and is based on the cumulative knowledge of cement and concrete chemistry obtained through research at NIST [76].

**Fig. 6:**
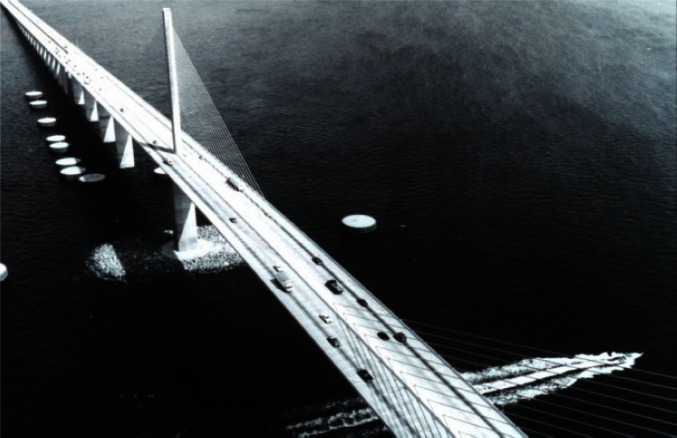
Structures of today, like the Sunshine Skyway bridge in Tampa, make use of advances in high-performance concrete.

Engineered cements from the raw materials to finished products are only possible given an improved understanding of the structure and properties of clinker phases, the influences of the calcium sulfate forms, and the effects of supplementary cementitious materials. Our ability to predict performance and modify cements for optimum performance may make engineered cements a material of the near future (Fig. 6). Our ability to perform these tasks is, in no small part, the product of the contributions of the NBS/NIST staff over the last 100 years to cement and concrete research.

## 5. Dimension Stone

In 1880 the Census Office and the National Museum in Washington, DC conducted a study of building stones of the United States and collected a set of reference specimens from working quarries. This collection was merged with the Centennial Collection of U.S. building stones that were first displayed at the 1876 Centennial Exhibition in Philadelphia. Descriptions of producing quarries, commercial building stones, and their use in construction across the country were compiled and reported in the 10th census of the United States in 1880 [77]. This collection of stones, now augmented with building stones from other countries, was placed on display in the Smithsonian Institution.

In 1942, a committee was appointed to consider whether any worthwhile use could be made of the collection. It was decided that a study of actual weathering on such a great variety of stone would yield valuable information. A plan was developed for building a test wall at the National Bureau of Standards (NBS) as a cooperative study between NBS and ASTM Committee C 18 on Building Stone. Subsequently, in 1948 a test wall was constructed at the NBS site in Washington DC under the direction of D. W. Kessler [78].

**Fig. 7:**
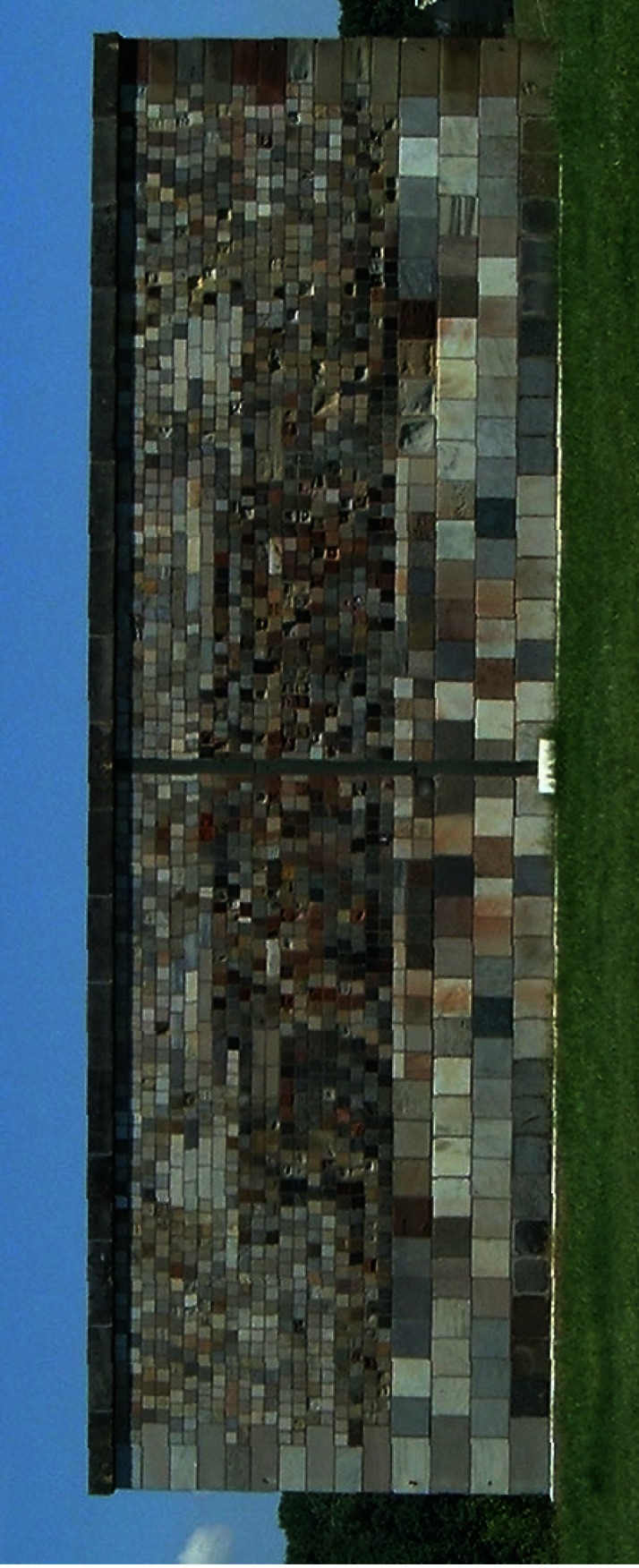
The Stone Test Wall is one of the largest collections of building stone maintained for over 50 years as a weathering experiment.

The move of NBS to its present site in Gaithersburg, Maryland in the middle 1960s and the occupancy of the old NBS site by the University of the District of Columbia placed the wall in jeopardy. Inorganic Building Materials Group Leader, James R. Clifton, arranged to move the wall intact in May 1977 to its present site at NIST in Gaithersburg, MD (Fig. 7).

The purpose of the stone test wall is to study the performance of stone subjected to weathering. While stone is generally considered an inherently durable material, some stones do not perform equally well under the range of exposures encountered in construction. Weathering phenomena may be physical; through frost action or thermal expansion, chemical; through dissolution, hydrolysis, oxidation, or biological; through acid secretions from lichen growth.

Over 30 distinct types of stones are represented, some of which are not commonly used for building purposes. There are many varieties of the common types used in building, such as marble, limestone, sandstone, and granite. It contains 2352 individual samples of stone, 2032 of which are domestic stone from 47 states, and 320 stones from 16 foreign countries. The wall faces south and is constructed as a mirror-image, with the left-wall stone set in lime mortar and the right-wall stone set in portland cement mortar. Archive specimens for most of the stone have been preserved indoors.

The wall provides a rare opportunity to study the effects of weathering on different types of stones, with the climatic conditions being the same for all stones. It offers a comparative study of the relationships between mineralogy, texture and the durability of many common building stones that have been used in monuments, commercial, and government buildings. Also, the wall has served to preserve a valuable collection of building stone and should be useful as a reference for builders in identifying the kinds of stone that may be locally available. As the wall passes half a century in age, interesting degradation features have been observed [79]. A web site has been created (http://stonewall.nist.gov) to document the collection of both the archive and weathered stone.

## 7. Summary

The continuous theme for the Building Materials Divisions research is developing an improved understanding of materials performance and advancing construction materials science and technology. Their work has improved our construction materials knowledge through applications of measurement technology, service life prediction, and standards development for evaluation of a wide range of construction materials.
